# Following the Money: The Use of Funds for Undergraduate Medical Education in Psychiatry

**DOI:** 10.1192/bjo.2026.11315

**Published:** 2026-06-30

**Authors:** Jennifer Hanks, Sophia Bashir, Sherlie Arulanandam

**Affiliations:** 1 Avon and Wiltshire Partnership NHS Trust, Bristol, United Kingdom; 2 Bristol Medical School, Bristol, United Kingdom

## Abstract

**Aims:**

The Medical Education Tariffs paid to healthcare providers to cover the costs of hosting and training medical students on clinical placements are not a direct payment for teaching quality, but a contribution to provider costs. In England, the 2024/25 allocations were set at £34,355 per medical student. Trusts are required to submit annual accountabilityreports to regulatory bodies on the use of this funding. However, accessing these reports or obtaining clarity on their content is very challenging and the quality assurance provided remains poor.

**Methods:**

We sent a Freedom of Information request to all the mental health trusts and health boards in the UK and Northern Ireland in December 2025 to explore their management of the undergraduate medical education funding and their adherence to reporting tools in place.

**Results:**

The overall response rate was 89% from 70 trusts and health boards across the four nations that offer psychiatric placements to medical students.

England 87% response.

Wales 100% response.

Scotland 85% response.

Northern Ireland 80% response.

Ringfencing of funding specifically for psychiatric undergraduate education happens in 27% of responders across Scotland, Wales and Northern Ireland and 82% of responders in England.

Specific records on the use of funding are kept in 59% of responders across Scotland, Wales and Northern Ireland and 77% responders in England, but less than 5% were able to provide a full breakdown of costs.

Employment of Clinical teaching fellows was the most consistent use of the funds across the four nations.

Reporting tools are used by 90% of responders in Scotland, Wales and Northern Ireland and 69% of responders in England.

Reports are available for public viewing in just 23% responders in Scotland, Wales and Northern Ireland and 10% of responders in England.

**Conclusion:**

There was significant variation in the use and monitoring of funds between the four nations. All providers need to fulfil their statutory obligation to provide detailed reports to the designated regulatory bodies on their management of the undergraduate education tariff. Effective, transparent management of the education budget is crucial to support the development of the future health workforce as discussed in the NHS 10-year plan. The College Undergraduate Education Forum is setting up a working group to develop guidance on the use of tariffs and will continue to encourage transparency and clarity on the process.

